# Evaluation of classification performance for six types of fundus diseases in OCT images based on multi-source training strategy

**DOI:** 10.3389/fmed.2026.1775911

**Published:** 2026-03-05

**Authors:** Biao Guo, Daqing Wang, Zhuo Zhao, Wenchao Liu, Jia Hou, Ruilin Liang, Lijuan Zhang

**Affiliations:** 1 Netchina Huaxin Technology Co., Ltd., Taiyuan, Shanxi, China; 2 Shanxi Eye Hospital, Taiyuan, Shanxi, China; 3 Shanxi Medical University, Taiyuan, Shanxi, China; 4 The 985th Hospital of the Joint Logistics Support Force of the Chinese People's Liberation Army, Taiyuan, Shanxi, China

**Keywords:** data fusion, deep learning, multi-category classification, OCT, retinal diseases

## Abstract

**Objective:**

Currently, publicly available Optical Coherence Tomography (OCT) datasets are commonly plagued by limited coverage of disease categories, scarce samples and severe class imbalance, which leads to insufficient generalization ability of deep learning models in real-world clinical settings. This study aims to construct a high-quality OCT dataset encompassing six key types of fundus lesions and normal controls, and to systematically evaluate the improvement effect of training strategies for multi-source data fusion on the performance of multi-class classification.

**Methods:**

We integrated local clinical data from Shanxi Eye Hospital with the latest public dataset OCTDL to establish a combined dataset. This dataset consists of 6,165 images, covering seven categories: age-related macular degeneration (AMD), diabetic macular edema (DME), retinal artery occlusion (RAO), retinal vein occlusion (RVO), epiretinal membrane (ERM), vitreomacular interface disease (VID), and normal controls (NO). On this basis, six representative deep learning architectures were selected, and two training paradigms were compared under unified experimental settings: (1) Training exclusively on open-source OCTDL data (S1); (2) Joint training using both local data and OCTDL data (S2). All models were evaluated on the identical OCTDL test set. A comprehensive analysis was conducted using multi-dimensional metrics including accuracy, weighted F1-score, class-specific recall, and area under the curve (AUC), with a particular focus on the misdiagnosis rate.

**Results:**

The S1 strategy exhibited significantly limited model recognition capability due to the extremely small sample sizes of certain categories. In contrast, the S2 strategy markedly improved the overall performance of the models. Confusion matrix analysis demonstrated that ViT-Base achieved the optimal performance under the S2 strategy: the accuracy reached 93.61%, the misdiagnosis rate of RAO was reduced to 0%, the misdiagnosis rate of AMD was controlled at 1.34%, and the misdiagnosis rate of RVO decreased from 14.89 to 8.51%.

**Conclusion:**

Multi-source data fusion serves as an effective approach to enhance the robustness of OCT multi-category classification models, and it can notably strengthen the recognition capability for certain diseases in particular. This study not only verifies the universal benefits of this strategy but also reveals the critical impact of model selection on the transfer learning effect.

## 1 Introduction

As a non-invasive imaging technique, optical coherence tomography (OCT) has emerged as a core tool for the clinical diagnosis of macular and retinal diseases in ophthalmology. In recent years, deep learning has exhibited tremendous potential in the task of automatic OCT image classification, and its performance in identifying common diseases such as age-related macular degeneration (AMD) and diabetic macular edema (DME) has already reached or even approached the level of clinical experts. However, most publicly available OCT datasets currently cover only a limited range of disease categories. Such data bias restricts the generalization capability of the trained models in real clinical scenarios; when confronted with unseen lesion types, the models tend to misclassify them into known categories or generate predictions with low confidence, thereby undermining the reliability of clinical decision-making. In addition, datasets from a single institution often suffer from issues such as small sample sizes and low device heterogeneity, which are insufficient to support the adequate training of complex models.

To address the aforementioned challenges, this study proposes and constructs a novel dataset comprising six types of OCT-related lesions and normal controls, which includes: Age-Related Macular Degeneration (AMD), Diabetic Macular Edema (DME), Retinal Artery Occlusion (RAO), Retinal Vein Occlusion (RVO), Epiretinal Membrane (ERM), Vitreomacular Interface Disorder (VID), and Normal (NO). On this basis, we systematically evaluate the impact of two training paradigms on model performance: (1) independent training with open-source data; (2) joint training with concatenated local and open-source data. Six representative deep learning architectures are selected for fair comparison under a unified experimental setup, with a focused analysis on the improvement in the models’ recognition performance for different categories of retinal diseases.

The main contributions of this study are summarized as follows:

1. Construct a dataset encompassing six clinically significant OCT lesions and normal controls.
2. Systematic evaluation of the enhancement effect of multi-source data fusion on OCT multi-category classification tasks.
3. Provision of comprehensive benchmark experiments involving both CNN and Transformer architectures, which can serve as a reliable baseline for future research.

## 2 Related work

### 2.1 Related work on OCT datasets

The development of open-access optical coherence tomography (OCT) datasets serves as a critical cornerstone for translating intelligent retinal diagnosis from the laboratory to clinical practice. Over the past decade, OCT datasets have undergone a systematic transformation: from extreme scarcity to relative abundance, from small-scale single-disease samples to clinical-grade multi-disease cohorts, and from single-fluid labeling to fine-grained multi-task annotations.

In 2015, Chiu et al. made public 110 B-scans from 10 patients with severe diabetic macular edema (DME), along with a pixel-level gold standard for fluid segmentation and seven-layer retinal layer segmentation (29). In 2016, Wu et al. released an intraretinal fluid (IRF) dataset consisting of 30 OCT volumes from multiple manufacturers (Cirrus, Spectralis, and Topcon). These volumes were independently annotated by multiple annotators, and for the first time, inter-annotator agreement was systematically evaluated, pioneering a framework for multimanufacturer compatibility and standardized assessment (30). In 2017, Rashno et al. expanded and made public the local UMN dataset based on the Duke and Optima datasets. Collectively, these three datasets became a standard open-source resource for early research on DME fluid segmentation (31).

The year 2018 marked a breakthrough era. Kermany et al. released approximately 110,000 OCT images, covering four categories: normal controls, choroidal neovascularization (CNV), DME, and drusen (32). Launched in the same year and formally published in 2019, the RETOUCH Challenge provided annotations for three types of fluid (IRF, subretinal fluid (SRF), and pigment epithelial detachment (PED)). It included 70 OCT volumes from three mainstream devices and two clinical centers, and was widely recognized as the gold standard for fluid segmentation between 2018 and 2022 (35). Although not fully made public, Schlegl et al. trained a fully automatic fluid detection model using 1,200 clinical OCT volumes (400 cases each of AMD, DME, and RVO), demonstrating the prototype of a clinical-scale large dataset (34).

Between 2019 and 2020, datasets advanced toward refinement for single diseases and single lesions. Specialized datasets were successively released, such as those for central serous chorioretinopathy (CSC) with SRF/neuroretinal detachment (NRD) (37, 38, 40), PED (41), and the AI Challenger Macular Edema Competition (focusing on SRF and PED) (39). These datasets promoted the design of network architectures tailored to specific lesions, such as multi-scale and attention-based mechanisms.

After 2020, small-to medium-sized yet high-quality open-access datasets continued to emerge. Gholami et al. published the OCTID dataset, which contained over 500 high-resolution images covering normal controls, macular hole (MH), AMD, CSR, and diabetic retinopathy (DR), along with 25 images with a gold standard for layer segmentation (33). He et al. provided fine-grained segmentation data for eight retinal layers from 35 patients with multiple sclerosis (36). In 2024, Kulyabin et al. released the OCTDL dataset (42), which included more than 2,000 clinical images from Optovue Avanti, covering six categories: AMD, DME, ERM, RAO, RVO, and VID.

### 2.2 Applications of deep learning in OCT classification

In recent years, significant achievements have been made in the research on retinal disease classification. In 2017, Xu et al. proposed an improved supervised classification method for retinal arteriovenous images. By applying intra-image regularization and inter-subject normalization, the method captured discriminative features distinguishing arteries from veins using novel feature descriptors, achieving an overall accuracy of 0.923 on the DRIVE dataset, which provides a potentially important tool for the early diagnosis of multiple diseases (1).

In 2018, S. Najeeb et al. developed an algorithm for detecting regions of interest (ROIs) from retinal OCT scans, adopting a single-layer convolutional neural network (CNN) architecture for classification. After training on an open-source retinal OCT dataset, the algorithm demonstrated feasible classification accuracy (2).

In 2019, Z. Gao et al. trained a deep convolutional neural network model to grade the severity of diabetic retinopathy (DR) from fundus images, achieving an experimental accuracy of 88.72%. When deployed on a cloud computing platform, the model reached a consistency rate of 91.8% with ophthalmologists, verifying the effectiveness of deep neural networks for the automatic diagnosis of diabetic retinopathy (3). In the same year, L. Fang et al. proposed a Lesion-Aware Convolutional Neural Network (LACNN) for retinal OCT image classification. By guiding the CNN with retinal lesion information to simulate the diagnostic reasoning process of ophthalmologists, the model validated its effectiveness and efficiency on a clinical OCT dataset (4). Perdomo O et al. presented a deep learning model named oct-net, which extracts features from optical coherence tomography volumes to classify diabetes-related retinal diseases. The model achieved an accuracy of 93% and an area under the receiver operating characteristic curve (AUC) of 0.99 on public datasets, while also providing interpretable clinical information (5). Alqudah AM proposed a novel automatic convolutional neural network architecture for a multi-class classification system based on spectral-domain optical coherence tomography, attaining an overall accuracy of 95.30% for the classification of various retinal diseases (6).

In 2020, El-Hag NA et al. put forward a retinal image classification framework that performs classification through fuzzy preprocessing, image segmentation, gradient processing, and other steps, and additionally employs a convolutional neural network (CNN) to distinguish between normal and abnormal cases (7).

In 2021, J. Kim and L. Tran proposed deep learning models for four-category classification of patients’ OCT images. Combinations of different models exhibited high accuracy, sensitivity, and specificity, showing potential as a second reader for ophthalmologists (8). Yoo TK et al. demonstrated that few-shot learning using generative adversarial networks (GANs) can enhance the applicability of deep learning in the OCT diagnosis of rare retinal diseases, with the proposed model outperforming traditional methods (9). Ho E et al. trained multiple CNN architectures using a multi-disease retinal fundus image dataset to predict and classify pathologies, and the ensemble network achieved an AUROC score of 0.9613 for disease screening (10). Xu L et al. proposed a hybrid attention mechanism for classifying retinal lesion images, achieving high classification accuracy on public OCT datasets (11). Sunija et al. designed a classifier based on deep neural networks to identify severe retinal lesions from OCT images, which not only realized high accuracy but also had advantages in real-time applications (12).

In 2022, Mohan R et al. developed a new convolutional neural network architecture called MIDNet18 for retinal disease classification, reaching an accuracy of 98.86%, which outperformed other standard CNN models (13). Esfahani EN et al. presented a convolutional neural network method guided by edge convolution layers for the automatic classification of OCT images, with an average accuracy of 99.43% (14). Asif S et al. adopted a deep residual network for the classification of four types of retinal diseases, achieving an overall classification accuracy of 99.48%, superior to existing approaches (15). Playout C et al. evaluated the performance of several Transformer models for retinal disease classification and proposed a focused attention mechanism to generate interpretable predictions, verifying that Vision Transformers (ViTs) have higher interpretability (16).

In 2023, R. Singh et al. employed the pre-trained VGG-16 architecture for retinal disease identification, attaining an accuracy of 85% (17). Rodriguez MA et al. proposed a multi-label classification system for detecting multiple retinal diseases, where the Transformer-based model outperformed state-of-the-art methods in terms of AUC scores for disease detection and classification (18). Daich Varela M et al. conducted a review of studies that use artificial intelligence to provide decision support for the diagnosis and classification of retinal diseases (19). Peng J et al. proposed a multi-scale denoising residual convolutional network for retinal disease classification, which achieved high classification accuracy and strong noise robustness on public datasets (20). He J et al. presented an interpretable Swin-Poly Transformer network for retinal OCT image classification, reaching an accuracy of 99.80% and an AUC of 99.99% (21).

In 2024, Vishal et al. proposed a specific retinal disease detection model combining convolutional neural networks and support vector machines, with overall accuracy, recall, and F1-score all exceeding 93% (22). B. D. Nath and S. Afroge applied transfer learning and fusion techniques for the classification of various ophthalmic diseases, achieving high accuracy on datasets and surpassing existing methods (23). Zhao J et al. proposed a framework combining ResNet and Transformer for multi-label classification of retinal diseases, which outperformed other multi-label classification methods on public datasets (24). Liu X et al. introduced a framework for quantifying biomarkers in optical coherence tomography and optical coherence tomography angiography images for retinal diseases, obtaining high accuracy and F1-score in disease classification tasks (25). Laouarem A et al. proposed a hybrid model for multi-label classification, which combines the advantages of convolutional neural networks and Vision Transformers, achieving high accuracy and computational efficiency across multiple datasets (26). Akça S et al. proposed multiple Vision Transformer algorithms for retinal disease detection, with Mobile-ViT showing superior performance in classification accuracy (27). Prabha AJ et al. developed a lightweight hybrid learning network for retinal disease OCT classification, which achieved high accuracy and AUC values for the classification of multiple retinal diseases (28).

## 3 Methods

### 3.1 Dataset construction

In this study, a multi-device ophthalmic optical coherence tomography (OCT) image dataset was constructed to support the intelligent diagnosis and analysis of various common retinal diseases. The images in this dataset were acquired using two clinically prevalent devices, namely Heidelberg Spectralis OCT and VG 200D (Intalight Ltd., China), thus reflecting the challenge of device heterogeneity in real-world clinical scenarios. The dataset covers seven clinical phenotypes, including age-related macular degeneration (AMD), diabetic macular edema (DME), epiretinal membrane (ERM), normal control samples (NO), retinal artery occlusion (RAO), retinal vein occlusion (RVO), and vitreomacular interface disease (VID). Specifically, it consists of 1,915 images of AMD collected from 59 patients; 1,174 images of DME obtained from 78 patients; 786 images of ERM involving 88 patients; 204 normal control images sourced from 50 participants; 191 images of RAO contributed by 15 patients; 189 images of RVO related to 31 patients; and 306 images of VID derived from 34 patients. This dataset not only encompasses a wide range of ophthalmic diseases with distinct clinical significance but also takes device diversity into account, thereby laying a solid foundation for the development of ophthalmic artificial intelligence models with high robustness and favorable generalization capability.

The inclusion criteria for OCT images from Shanxi Eye Hospital were as follows: all images were obtained via linear OCT scans centered on the fovea centralis, with a scanning range covering a 20° × 20° macular area. All images were screened by two experienced attending ophthalmologists, and only those images that clearly exhibited the disease features described in this study were included. Images with controversial diagnostic findings were adjudicated by a senior chief ophthalmologist to determine their eligibility for inclusion. For images presenting features of two concurrent diseases (e.g., a DME patient with comorbid ERM), the images were classified solely under the category corresponding to the primary disease manifestation.

The core OCT features of age-related macular degeneration (AMD) are characterized by the distinctive destruction of retinal structures in the macular region. In the dry form of AMD, OCT imaging reveals scattered or confluent drusen (manifesting as hyperreflective elevations) beneath the retinal pigment epithelium (RPE). In the advanced stage, thinning or loss of the outer retinal layers and the RPE occurs, a condition known as geographic atrophy.

In the wet form of AMD, typical features include the presence of moderately to highly reflective fibrovascular proliferative lesions (polypoidal lesions or classic neovascularization) beneath the neurosensory retina and/or the RPE. These lesions are often accompanied by intraretinal or subretinal hyporeflective cystoid spaces (edema or exudation), as well as detachment of the neurosensory retina or the RPE. As the disease progresses, both forms of AMD can ultimately lead to structural disorganization, disruption of all retinal layers in the macular region, and subsequent scar formation (hyperreflective masses).

**Figure 1:**
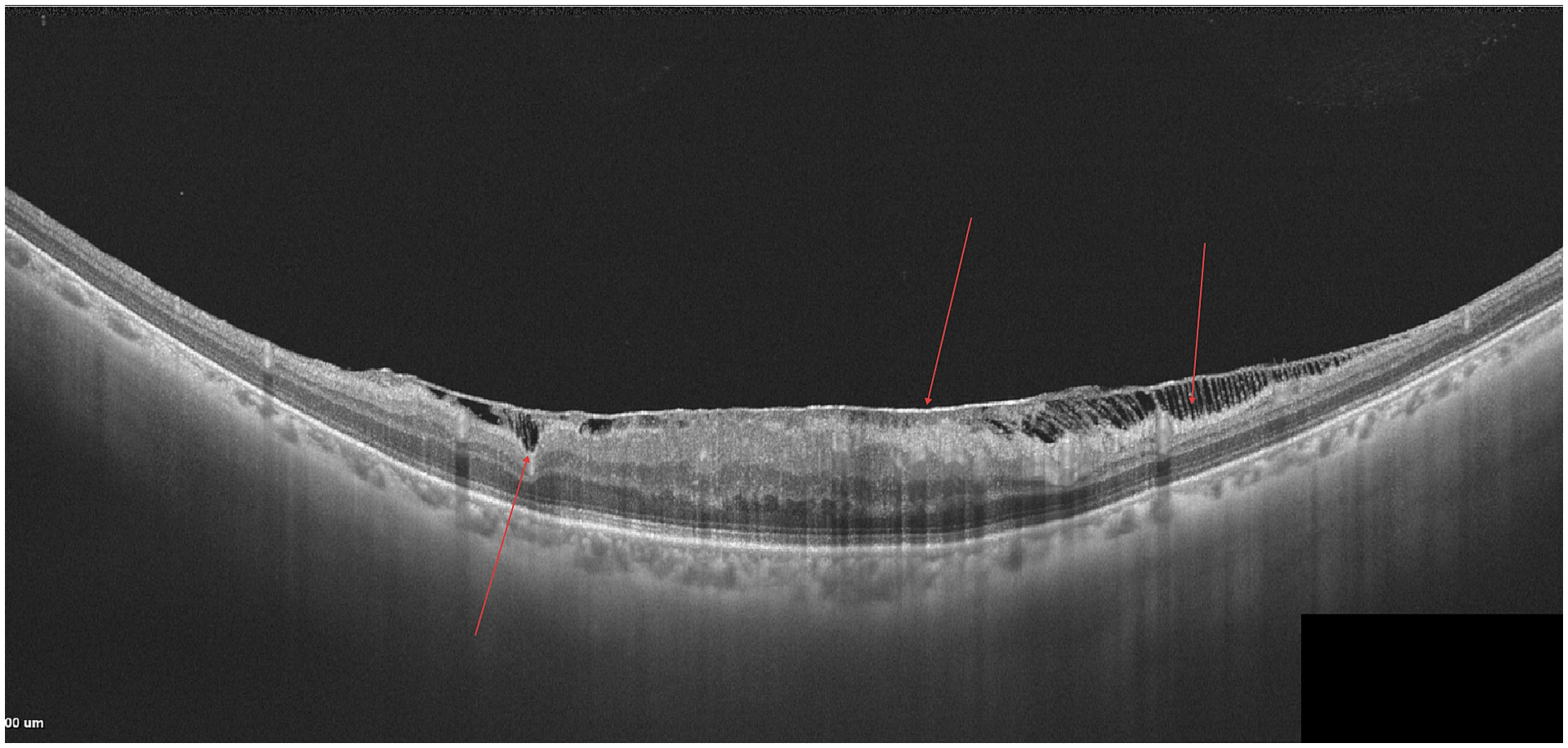
AMD.

As shown in Figure 1, a large area of moderately to highly reflective lesions and small hyporeflective cystoid spaces can be observed beneath the neurosensory retina in the macular region. The RPE layer appears discontinuous, with several punctate hyperreflective foci noted above it, and the interlaminar retinal structures are disorganized.

The core OCT feature of diabetic macular edema (DME) is intraretinal fluid accumulation, which is specifically manifested as cystoid macular edema. The hyporeflective cystoid spaces are predominantly located in the inner nuclear layer, and may also spread to the outer plexiform layer; in severe cases, these spaces coalesce into large cysts or lead to serous detachment of the neurosensory retina.

Characteristic associated signs include discrete hyperreflective spots corresponding to intraretinal hard exudates (often deposited in the outer plexiform layer/Henle’s fiber layer), retinal thickening, as well as disruption of the ellipsoid zone and irregularity of the external limiting membrane observed in chronic or severe cases—with the latter two indicating damage to photoreceptor structures. Some cases may be complicated by vitreomacular traction or epiretinal membrane.

**Figure 2:**
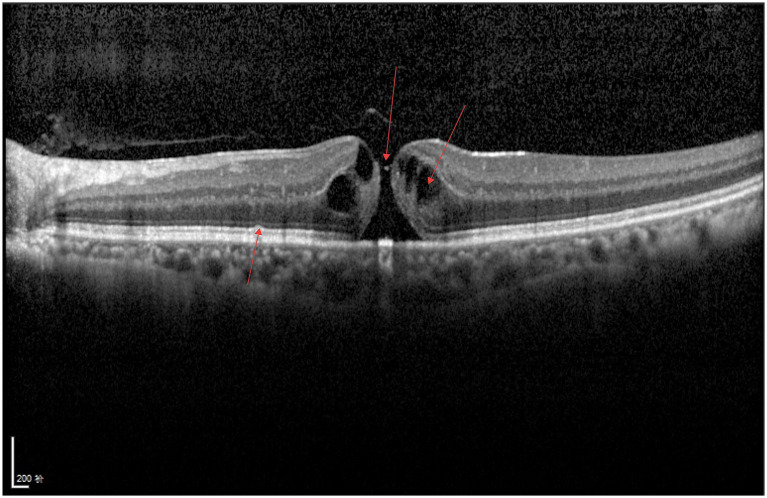
DME.

As illustrated in Figure 2, cystoid hyporeflective spaces and several scattered punctate or patchy hyperreflective foci can be observed between retinal layers, accompanied by structural disorganization. A large area of hyporeflectivity is present between the neurosensory retina and the retinal pigment epithelium (RPE) layer, the ellipsoid zone appears discontinuous, and hyperreflectivity is noted in the RPE layer at the fovea.

The core OCT feature of epiretinal membrane (ERM) is the presence of a continuous or discontinuous hyperreflective band on the surface of the internal limiting membrane (ILM) of the retina. This membrane can be locally adherent or extensively covering; its contractile traction induces morphological deformation of the underlying retinal structures, with typical manifestations including loss of the macular foveal contour, localized retinal thickening, and the possible formation of a pseudomacular hole or foveal wrinkles. Severe traction can lead to intraretinal edema (cystoid changes), neurosensory retinal detachment, and even a true full-thickness macular hole. The thickness of the membrane, the degree of hyperreflectivity, and the adhesion points between the membrane and the retina can be clearly visualized.

**Figure 3:**
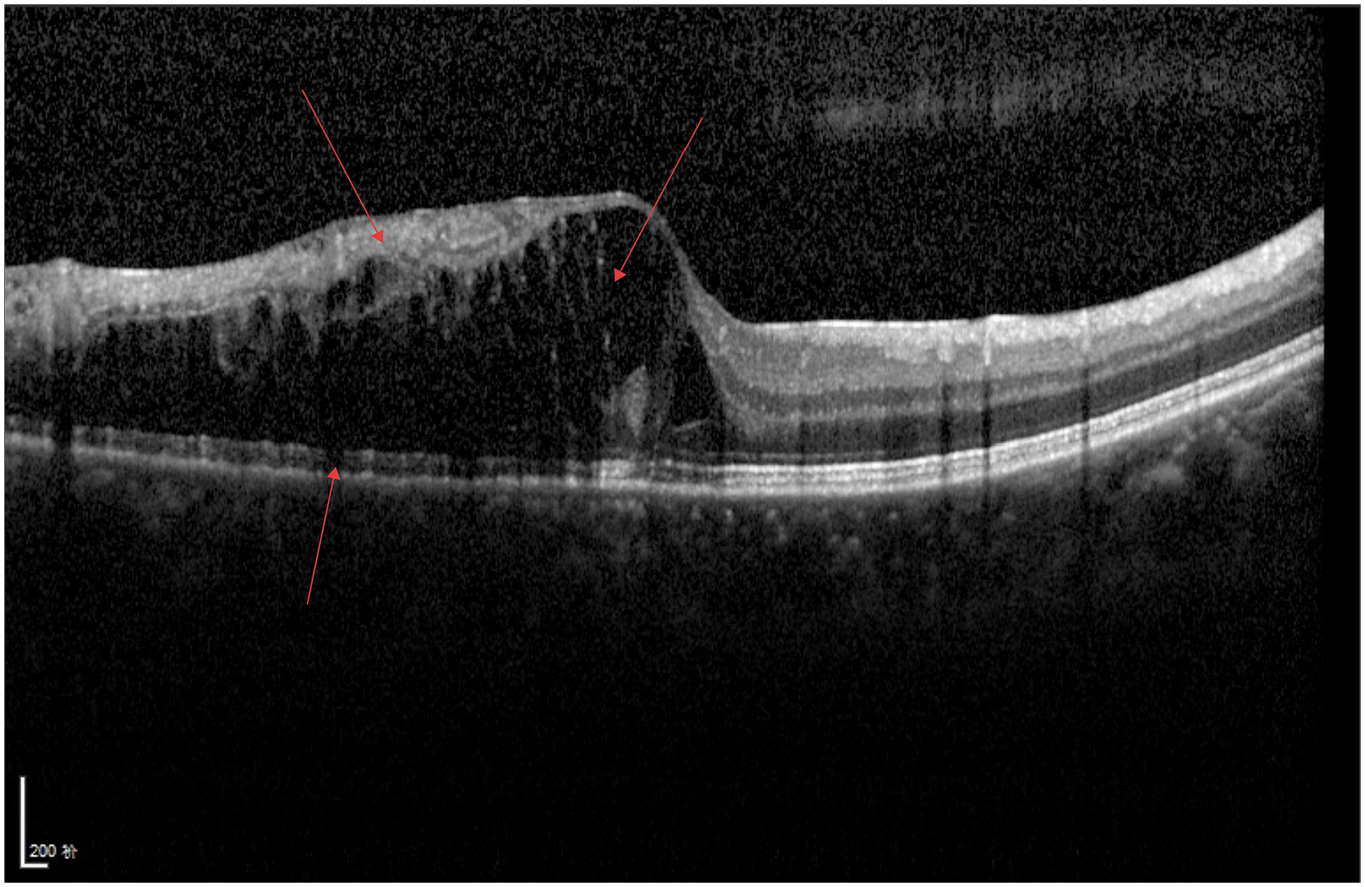
ERM.

As shown in Figure 3, a continuous hyperreflective band is visible on the surface of the retinal internal limiting membrane, the macular foveal contour disappears, the structures of all retinal layers are disorganized, traction adjacent to the fovea is evident, and fibrous bands can be observed.

The OCT imaging features of branch retinal artery occlusion (BRAO) are mainly characterized by varying degrees of thickening and edema of the neurosensory retina in the macular region, accompanied by the characteristic “outer retinal folds”. Specifically, the outer photoreceptor structures such as the external limiting membrane (ELM), ellipsoid zone (EZ), and cone outer segment tips (COST) exhibit disruption, disorganization, or wavy changes, often associated with hyporeflective cystoid or interlaminar fluid accumulation. The inner retinal layers are usually relatively intact; however, in the acute phase, small focal hyperreflective spots (likely migrated macrophages) can be observed beneath the internal limiting membrane in the affected area.

In the chronic or recovery phase, the outer retinal folds may alleviate or disappear, but permanent structural damage such as outer retinal atrophy, thinning, and EZ discontinuity may remain.

**Figure 4:**
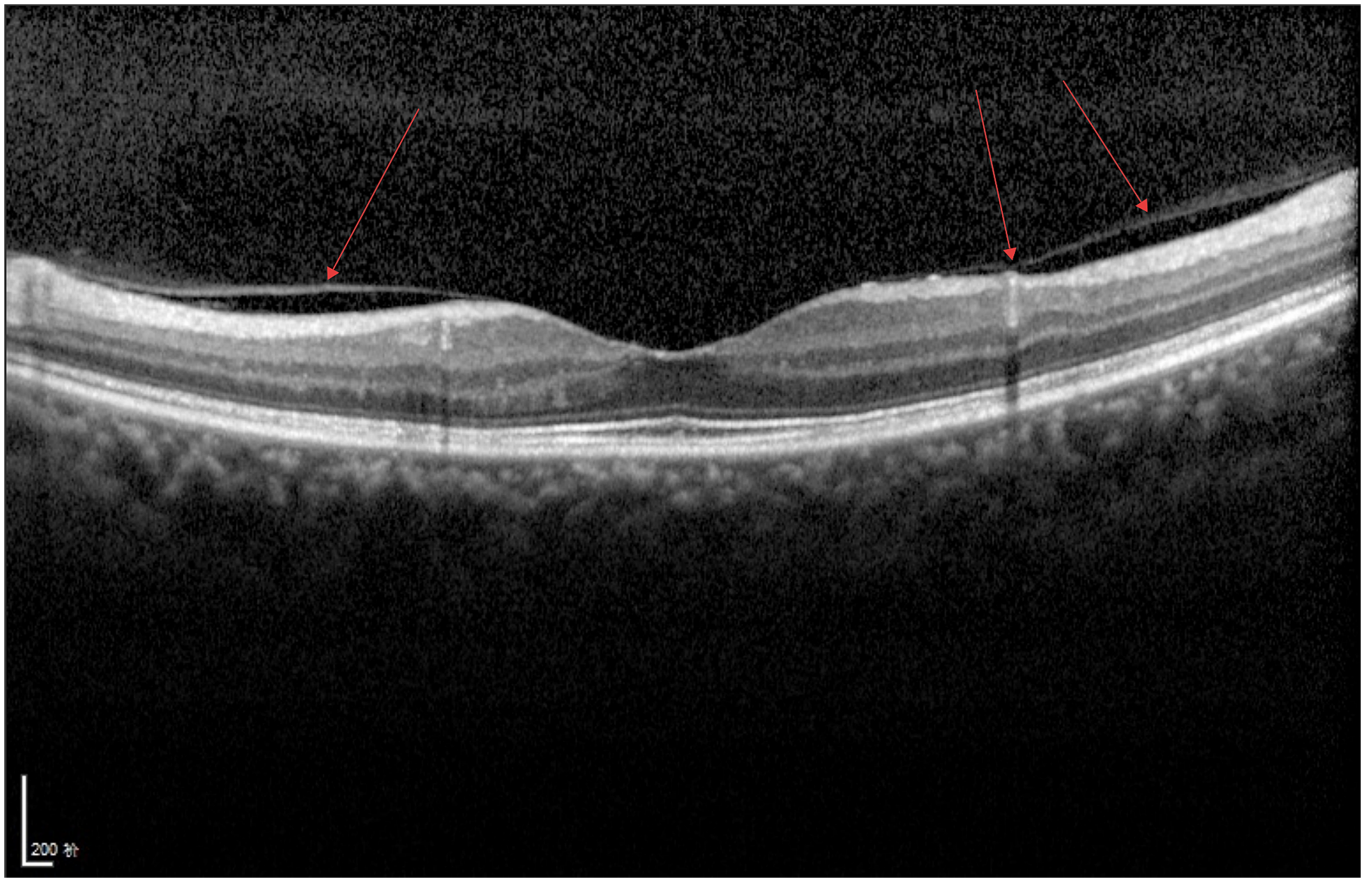
BRAO.

As shown in Figure 4, increased retinal thickness, enhanced reflectivity, blurred interlaminar structures, widened hyporeflective zones of the photoreceptors, and wavy changes of the RPE layer can be observed in the affected eye.

The OCT imaging features of central retinal artery occlusion (CRAO) vary across different disease stages, as detailed below:

In the acute phase (<24 h to several days), the core manifestation is diffuse, homogeneous hyperreflective thickening of the inner retinal layers caused by cytotoxic edema, forming the characteristic “middle retinal hyperreflective band.” The outer plexiform layer and the structures beyond it appear as relatively hyporeflective dark zones due to signal shadowing. In contrast, the outer retinal layers, which are vascularized by the choroidal circulation, remain intact in the early stage, presenting a clear boundary between the inner and outer retinal segments.

In the subacute phase (several days to weeks), the hyperreflective inner layers begin to show vacuolization or cystoid changes, followed by the irreversible stage of inner retinal atrophy, characterized by progressive thinning and even disappearance of the ganglion cell layer and inner nuclear layer.

The definitive feature of the chronic phase (> 1 month) is overall retinal thinning with complete loss of inner retinal structures, while the outer retinal architecture remains relatively intact. This forms the classic “double-layer sign” described as “inner layer atrophy and outer layer survival.” At this stage, the macular fovea may exhibit an abnormal “pseudonormalization” appearance due to the loss of inner retinal tissue. In addition, secondary findings such as “subinternal limiting membrane hyperreflective foci” and varying degrees of “cystoid macular edema” can be observed.

**Figure 5:**
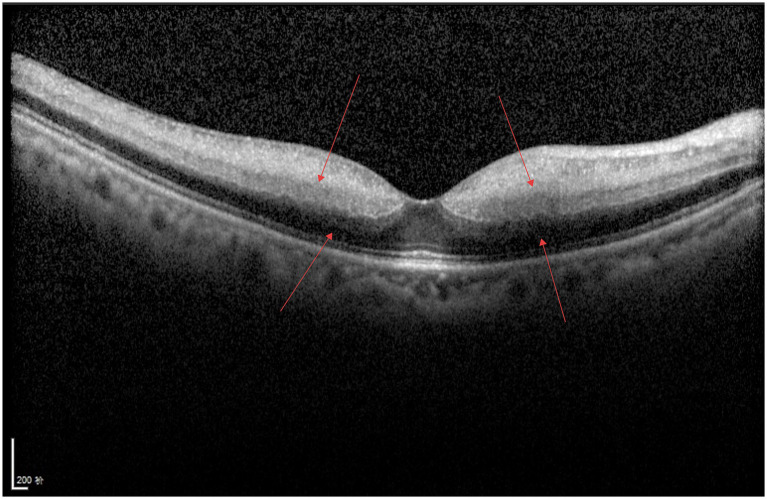
CRAO.

As shown in Figure 5, the inner retinal layers present diffuse hyperreflective thickening with blurred interlaminar structures, and the regions beyond the outer plexiform layer appear as hyporeflective dark zones.

The OCT imaging features of branch retinal vein occlusion (BRVO) are mainly characterized by quadrantal and asymmetric changes demarcated by the horizontal midline.

In the affected venous drainage area, diffuse thickening of the full retinal thickness is observed in the acute phase, accompanied by significant spongiform edema of the inner retina and cystoid macular edema (CME). The latter presents as cystoid hyporeflective cavities in the inner plexiform layer, outer plexiform layer, and inner nuclear layer. Due to capillary leakage, subretinal fluid (SRF) is often detectable.

In the chronic or ischemic phase of BRVO, the pathological changes are dominated by structural damage to the inner retina, manifesting as atrophy and thinning of the inner nuclear layer and inner plexiform layer, which form the characteristic “atrophic triangle.” Meanwhile, hyperreflective foci (HEP) or hard exudates may appear beneath the internal limiting membrane or in the deep retinal layers. In addition, as the superficial capillary plexus is damaged, hyperreflective hemorrhagic foci can be seen in the inner retina or preretinal space.

**Figure 6:**
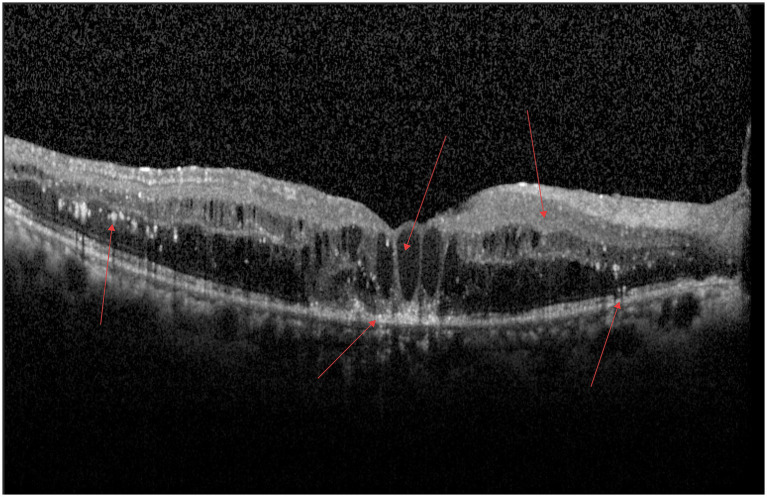
BRVO.

As shown in Figure 6, cystoid hyporeflective changes are visible beneath the neurosensory retina in the affected region, the inner retinal layers are thinned, the interlaminar structures are disorganized, and the ellipsoid zone and RPE layer are discontinuous.

In the acute phase, the core manifestation is diffuse thickening of the full retinal thickness, accompanied by prominent cystoid macular edema (CME). The cystoid cavities are predominantly concentrated in the inner nuclear layer and inner plexiform layer, and are often associated with subretinal fluid (SRF). In severe ischemic cases, extensive hyperreflective lesions corresponding to cotton wool spots and shadowing effects caused by deep hemorrhages can be observed in the inner retinal layers. Characteristic signs include optic disc edema, as well as numerous intraretinal hyperreflective foci (HEP) distributed along the major vascular arcades. The latter is associated with disruption of the blood-retinal barrier, lipoprotein exudation, and microglial aggregation.

In the chronic phase, the condition progresses to progressive diffuse atrophy and thinning of the full retinal thickness, with blurring of the inner retinal structures. Cystoid edema may persist or evolve into cystoid macular scars. In severe cases, secondary epiretinal membrane or macular hole may develop.

**Figure 7:**
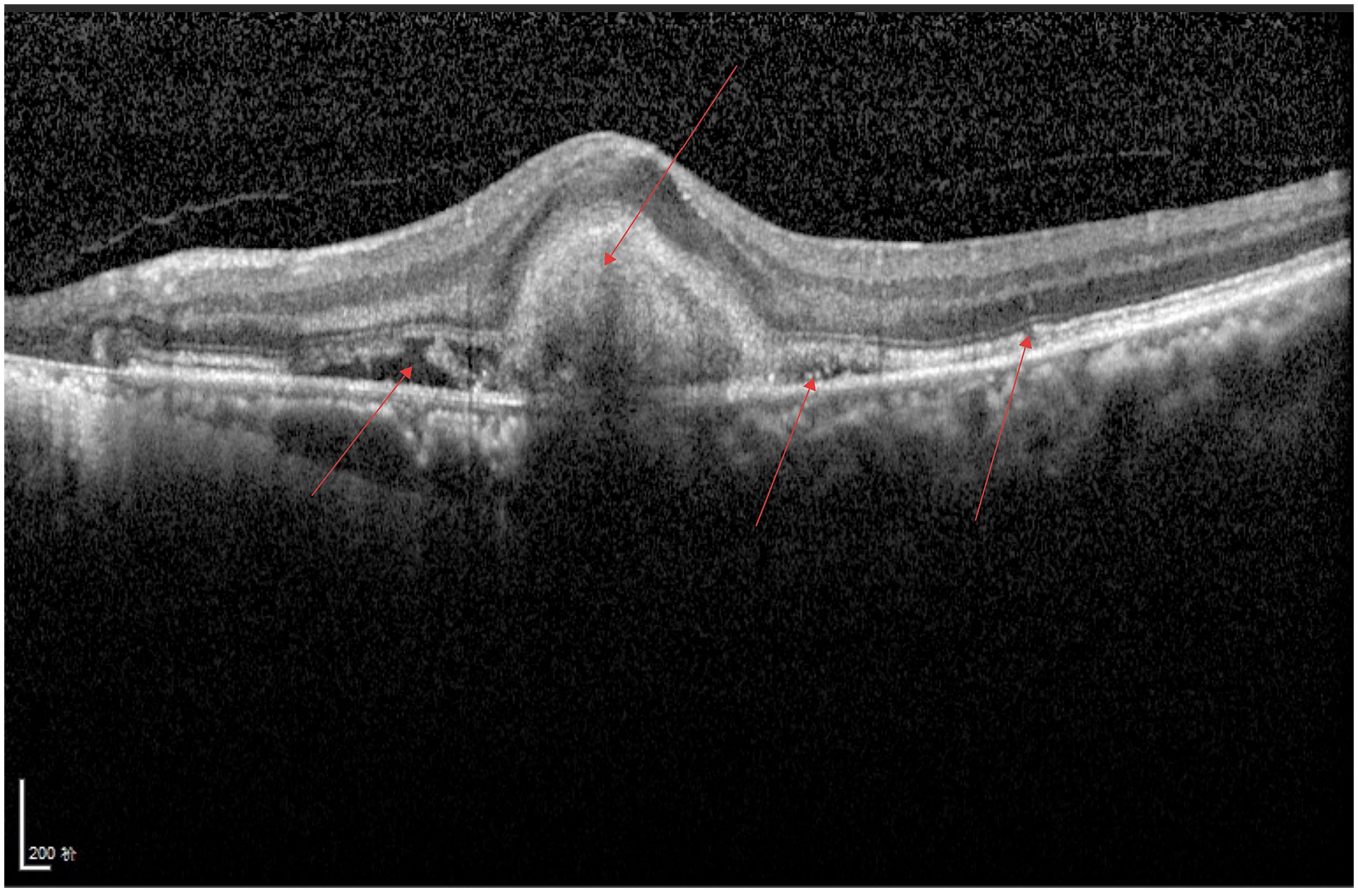
CRVO.

As shown in Figure 7, several cystoid hyporeflective regions are visible between the retinal layers, the ellipsoid zone is discontinuous, the neurosensory retina is detached from the RPE layer at the fovea, the ellipsoid zone disappears, and the structures of all retinal layers are disorganized.

Vitreomacular interface disease (VID) is a term used to describe a spectrum of disorders caused by the pathological processes of age-related posterior vitreous detachment (PVD). Ranging from simple separation in physiological PVD, to persistent traction in pathological vitreomacular traction (VMT), and ultimately to tissue defect in macular hole (MH), optical coherence tomography (OCT) serves as the gold standard for the diagnosis and differentiation of such diseases by clearly visualizing the anatomical relationship between the posterior vitreous cortex and the retinal internal limiting membrane (ILM).

The core OCT feature of PVD is the separation of the posterior vitreous cortex from the retinal internal limiting membrane. The typical manifestation is a smooth, continuous, thin moderately hyperreflective curved line visible anterior to the retina, representing the detached posterior vitreous cortex. This line can be clearly identified at the optic disc margin (the attachment site of the Weiss ring) and around the macular fovea (in the absence of adhesions), with a distinct, non-reflective dark zone—namely the vitreous cavity—formed between the line and the retinal surface. Based on the degree of detachment, PVD can be classified into complete PVD (the posterior cortex is detached entirely and appears suspended) and incomplete PVD (local adhesions persist, most commonly in the macular region or along the vascular arcades). If persistent adhesions with traction exist in the macular area, progression to vitreomacular traction syndrome (VMTS) may occur; OCT in such cases reveals localized retinal deformation, edema, or cystoid changes. The OCT manifestations of PVD form the fundamental basis for the assessment of vitreoretinal interface diseases.

**Figure 8:**
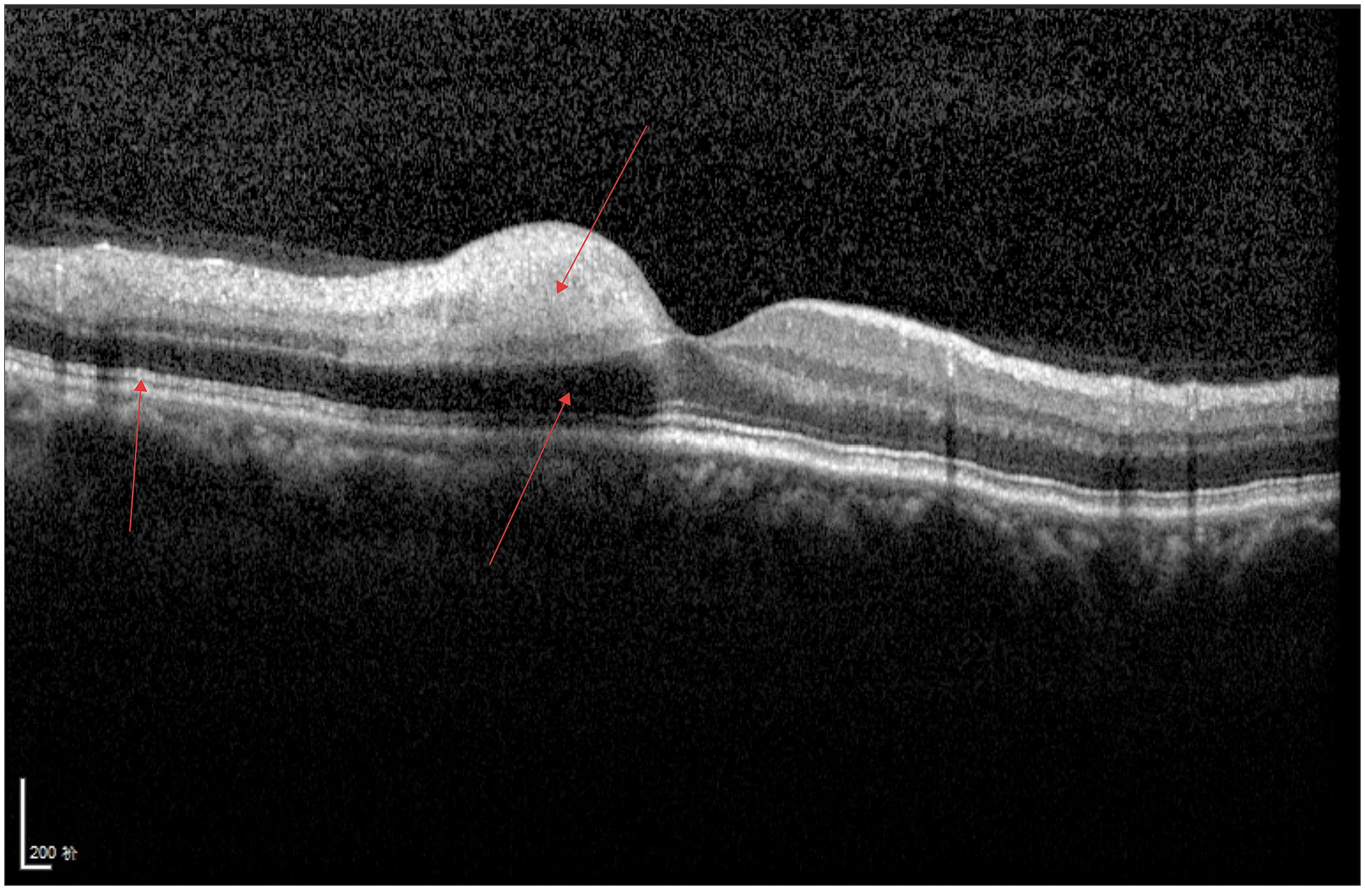
VID (PVD).

As shown in Figure 8, two smooth moderately hyperreflective curved lines are visible anterior to the retina, connected to the internal limiting membrane, with no obvious abnormalities observed in the interlaminar retinal structures.

The core OCT imaging feature of vitreomacular traction (VMT) is the abnormal adhesion and persistent traction between the posterior vitreous cortex and the internal limiting membrane (ILM) of the retina in the macular region. The typical manifestation is a moderately hyperreflective curved band representing the incompletely detached posterior vitreous cortex, which forms focal, vertical, or oblique adhesion points with the inner retinal surface at or adjacent to the macular fovea.

This pathological traction induces morphological deformation of the underlying retinal structures, with common signs including: loss or distortion of the macular foveal contour, localized thickening of the neurosensory retina, cystoid macular edema (hyporeflective cystoid cavities in the inner nuclear layer), and varying degrees of shallow detachment of the neurosensory retina. The tractional vitreous band usually appears taut, with distinct adhesion points to the retina—this is the key differentiating feature from physiological incomplete posterior vitreous detachment (PVD). Persistent traction can further lead to the formation of lamellar or full-thickness macular holes.

**Figure 9:**
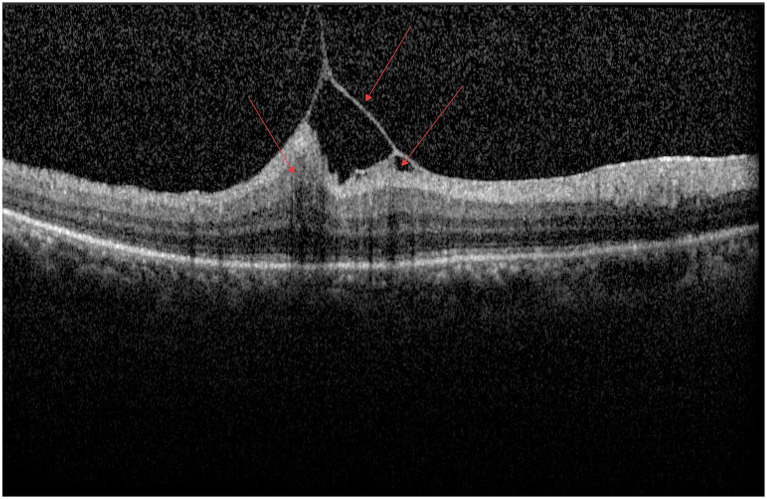
VID (VMT).

As shown in Figure 9, a hyperreflective band is visible anterior to the retina, connected to the internal limiting membrane. The macular foveal contour disappears, with cystoid hyporeflective changes present; localized deformation and thickening of the neurosensory retina are observed, along with structural disorganization of all retinal layers in the macular region.

The core OCT feature of macular hole (MH) is a full-thickness or partial laminar tissue defect of the neurosensory retina at the macular fovea. For full-thickness macular holes (FTMH), a complete discontinuity of the neurosensory retina can be observed, forming a hole with sharp margins. This lesion is often accompanied by edema and thickening of the retinal tissue at the hole’s edge (with the operculum possibly adhering to the posterior vitreous cortex or floating anterior to the hole), enhanced hyperreflectivity of the retinal pigment epithelium (RPE) at the hole’s base, as well as varying degrees of subneurosensory fluid or cystoid edema around the hole. Based on size and morphology, FTMH can be classified into small holes (≤250 μm), medium-sized holes (250–400 μm), and large holes (>400 μm).

Lamellar macular holes (LMH) are further divided into two subtypes: inner lamellar holes (characterized by the loss of only the inner retinal layers with intact outer retinal structures) and outer lamellar holes (manifesting as the loss of outer retinal layers accompanied by disruption of the external limiting membrane [ELM] and ellipsoid zone [EZ], with an intact internal limiting membrane [ILM]). For tractional macular holes, distinct signs of tangential traction caused by the vitreous cortex or epiretinal membrane can be identified.

**Figure 10:**
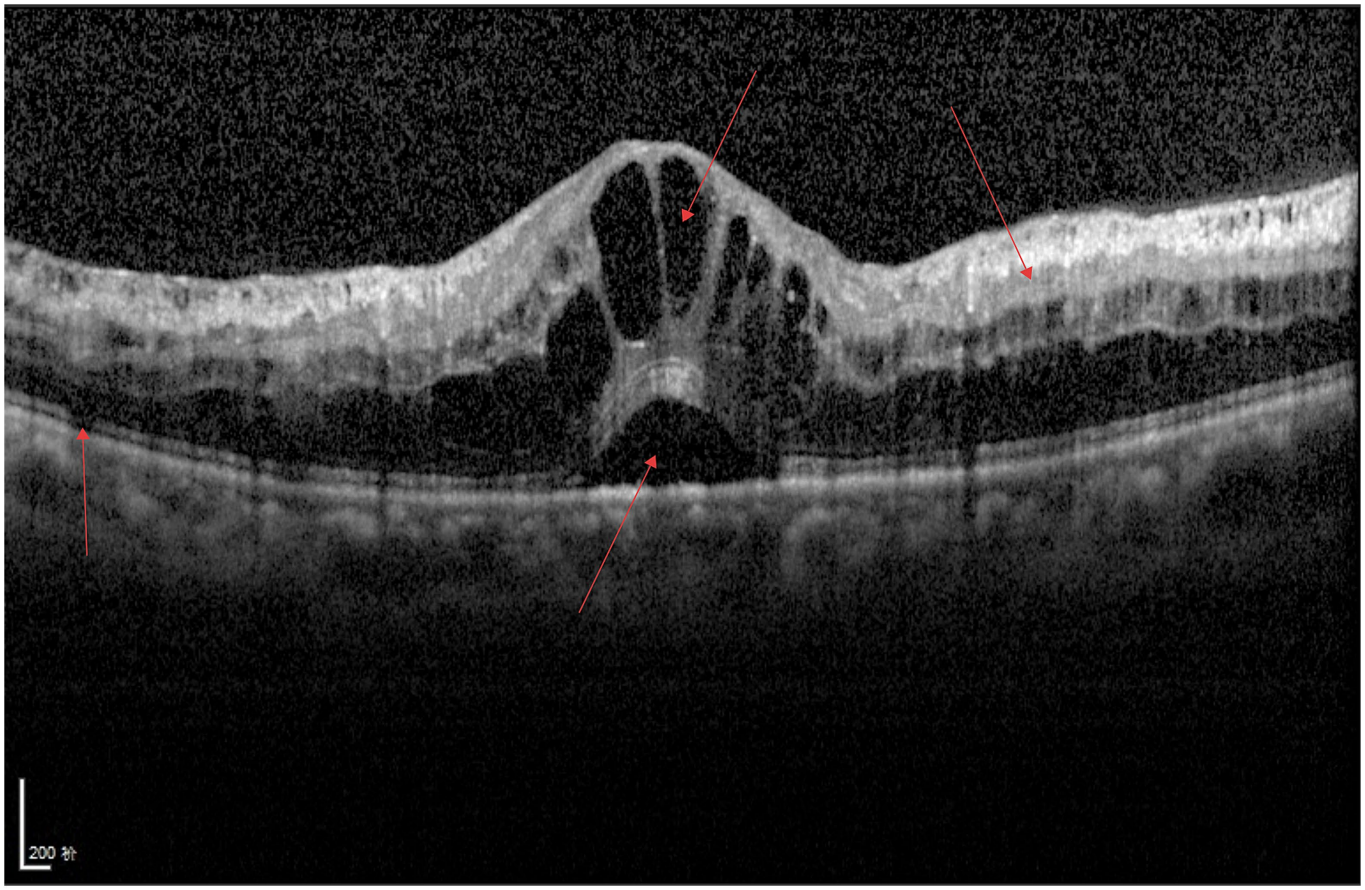
VID (MH).

As shown in Figure 10, a complete discontinuity of the neurosensory retina is visible at the macular fovea, with adjacent retinal thickening and cystoid hyporeflective regions of varying sizes. Localized elevation of the RPE layer and hyperreflectivity of the macular RPE layer are also observed.

In addition, we constructed a fused dataset by integrating clinical data from Shanxi Eye Hospital with the publicly available OCTDL dataset, aiming to further enhance data diversity and representativeness. OCTDL is a widely used open-source OCT image dataset, comprising 821 patients and 2,064 scans across 7 categories of fundus diseases. Among these categories, age-related macular degeneration (AMD) accounts for the largest proportion, with 421 patients and 1,231 images. The remaining categories include diabetic macular edema (DME, 107 patients), epiretinal membrane (ERM, 71 patients), normal controls (NO, 110 patients), retinal artery occlusion (RAO, 11 patients), retinal vein occlusion (RVO, 50 patients), and vitreomacular interface disease (VID, 51 patients), corresponding to 147, 155, 332, 22, 101, and 76 images, respectively.

**Figure 11:**
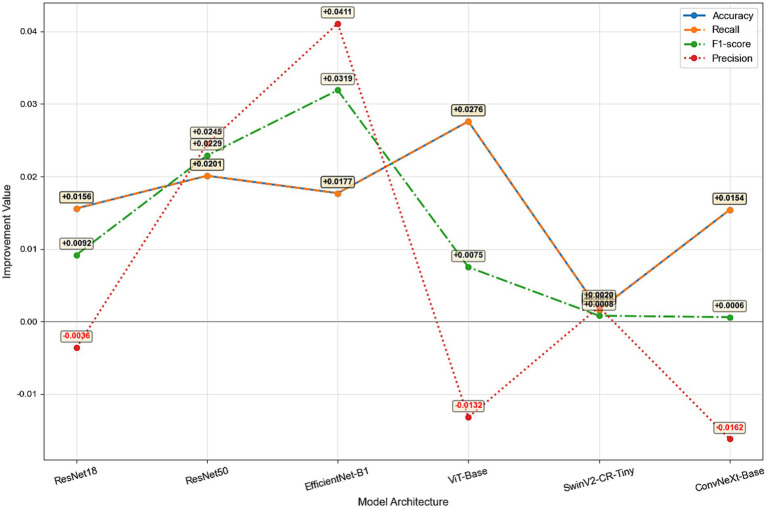
Distribution of patients across disease categories, and the number of cases per disease category.

By merging the two data sources, the final combined dataset consists of 1,176 patients and 6,165 OCT images, covering all 7 disease categories. This fused dataset not only significantly expands the sample size, but also combines the advantages of real-world clinical data (from Shanxi Eye Hospital) and open benchmark data (OCTDL). It demonstrates greater diversity and generalization potential in terms of patient distribution, device sources, and lesion manifestations. Figure 11 presents the patient count distribution and image count distribution across all disease categories for the two datasets.

This study has obtained ethical approval from the Ethics Committee of Shanxi Eye Hospital (Approval No.: SXYYLL-KSSC021). Since it is impossible to obtain written consent for this retrospective case picture study, and the pictures used in the study have been de-identified, covering up the patients’ private information. Moreover, privacy protection training has been provided for the personnel of the cooperating parties, and measures such as clearly defining the scope of data use have been taken, which can ensure the security of patients’ information.

### 3.2 Training strategies

In this study, we designed two training paradigms to systematically evaluate the generalization capability of models under different data sources. The first strategy (S1) involved training, validation, and testing exclusively on the publicly available OCTDL dataset, which covers 7 categories of ophthalmic diseases. The dataset was randomly partitioned into training, validation, and test sets at the patient level with a ratio of 60:10:30. Critically, all images from an individual patient were assigned to a single subset, thereby effectively avoiding data cross-contamination. The second strategy (S2) integrated private clinical data from Shanxi Eye Hospital with open-source OCTDL data during the training phase. Both datasets were uniformly mapped to the identical 7-category diagnostic label system, while the validation and test sets were strictly restricted to the pre-partitioned corresponding subsets from OCTDL. This experimental design ensured that S1 and S2 shared identical evaluation benchmarks, enabling a fair comparison between the two strategies.

The core advantage of this experimental framework lies in its focused assessment of model generalization. By anchoring the validation and testing processes entirely to the open-source dataset, we were able to objectively measure whether the introduction of external private data truly enhanced the model’s adaptability to new patients, rather than merely inducing overfitting to the data distribution specific to a single institution. Meanwhile, the patient-level data partitioning strategy effectively eliminated performance overestimation caused by the scattering of multiple images from the same patient across different subsets, thereby enhancing the clinical credibility of the experimental results. It is worth noting that in the implementation of the S2 strategy, we conducted rigorous label alignment and quality control on the data from Shanxi Eye Hospital, ensuring consistency with OCTDL in terms of semantic definitions and image standards. This step was intended to mitigate the risk of negative transfer arising from annotation discrepancies or device heterogeneity. Overall, this comparative paradigm not only balances reproducibility and real-world applicability but also provides a methodological reference for the development of multicenter medical imaging models.

**Figure 12:**
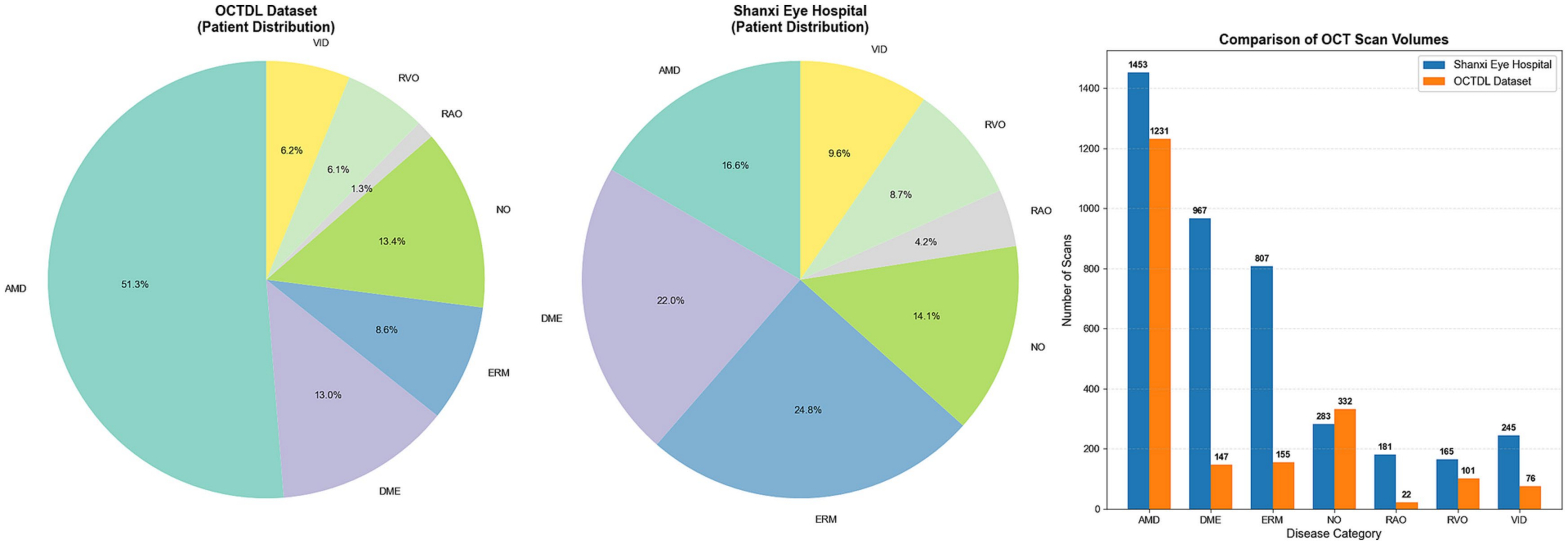
Distribution of data graph.

A comparative experiment was conducted using the two training paradigms described above. The training set for the S1 strategy was derived entirely from the OCTDL open-source dataset, consisting of 1,186 cases of AMD, 137 cases of DME, 111 cases of ERM, 279 cases of NO, 15 cases of RAO, 75 cases of RVO, and 67 cases of VID, totaling 1,870 cases. In contrast, the training set for the S2 strategy was formed by concatenating clinical data from Shanxi Eye Hospital with open-source OCTDL data, including 2,581 cases of AMD, 1,108 cases of DME, 918 cases of ERM, 562 cases of NO, 196 cases of RAO, 240 cases of RVO, and 312 cases of VID, totaling 5,917 cases. The two strategies shared identical validation and test sets, both sourced from the OCTDL dataset and partitioned at the patient level. The validation set comprised 491 cases of AMD, 49 cases of DME, 53 cases of ERM, 96 cases of NO, 3 cases of RAO, 28 cases of RVO, and 19 cases of VID, totaling 739 cases. The test set included 745 cases of AMD, 65 cases of DME, 72 cases of ERM, 156 cases of NO, 11 cases of RAO, 47 cases of RVO, and 38 cases of VID, totaling 1,134 cases. Figure 12 presents the statistical chart of data distribution.

In this experiment, all models were configured with identical training settings to ensure fair comparison: the number of training epochs was fixed at 100, and the batch size was set to 128. The Adam optimizer was adopted, with an initial learning rate of 0.0001 and a weight decay coefficient of 0.0005. A cosine annealing learning rate scheduling strategy was employed to smoothly reduce the learning rate in the later stages of training. Cross-entropy loss was uniformly used as the loss function across all models. The experiments were conducted on a hardware platform equipped with an NVIDIA GeForce RTX 4060 graphics card and a 12th Gen Intel^®^ Core™ i7-12700 processor. The development environment was built based on Python 3.12, with PyTorch 2.6.0 serving as the deep learning framework.

To comprehensively and objectively evaluate model performance, a multi-dimensional evaluation system was constructed in this study. Accuracy was used as the fundamental metric to measure the overall classification correctness. Meanwhile, Precision and Recall were introduced to assess the ability to control false positives and the capacity to identify false negatives, respectively. On this basis, the F1-score, as the harmonic mean of the two metrics, effectively addressed the issue of class imbalance. In addition, the area under the receiver operating characteristic curve (AUC) comprehensively reflected the model’s ability to distinguish between positive and negative samples at different thresholds and was insensitive to class distribution. Finally, the confusion matrix provided fine-grained classification results, clearly presenting the distribution of true positives (TP), false positives (FP), false negatives (FN), and true negatives (TN) across all categories, which facilitated the identification of the model’s performance shortcomings on specific categories. This comprehensive evaluation system ensured a thorough and in-depth analysis of model performance.

### 3.3 Model selection

In this study, seven representative deep learning architectures were selected for systematic comparison, covering two mainstream model paradigms: convolutional neural networks (CNNs) and Vision Transformers. Specifically, the CNN family included ResNet18, ResNet50, and EfficientNet; the Transformer family comprised Vision Transformer, Swin Transformer, and ConvNeXt.

To ensure the consistency of experimental conditions, the input image size of all models was uniformly adjusted to 224 × 224, and the models were initialized with pre-trained weights. This setup not only helped accelerate model convergence but also laid the foundation for a fair comparison of different architectures under the same benchmark.

## 4 Experimental results

### 4.1 Resource identification initiative

In this study, all models were evaluated on the identical test set (OCTDL, with a total of 1,134 cases) to enable a fair comparison of performance differences between the two training strategies: S1 and S2. Table 1 summarizes the final performance metrics of the six representative model architectures on the test set, including Accuracy, F1-score, AUC, Precision, and Recall.

**Table 1:** Performance metrics of six representative model architectures on the test set.

Model type	Training strategy	Accuracy	F1-score	AUC	Precision	Recall
ResNet18	S1	0.8855	0.9029	0.9881	0.9342	0.8855
ResNet18	S2	0.9011	0.9121	0.9839	0.9306	0.9011
ResNet50	S1	0.8863	0.8842	0.9778	0.8906	0.8863
ResNet50	S2	0.9064	0.9071	0.9844	0.9151	0.9064
EfficientNet-B1	S1	0.8875	0.8842	0.9783	0.8928	0.8875
EfficientNet-B1	S2	0.9052	0.9161	0.9867	0.9339	0.9052
ViT-Base	S1	0.9085	0.9242	0.9883	0.9477	0.9085
ViT-Base	S2	0.9361	0.9317	0.9887	0.9345	0.9361
SwinV2-CR-Tiny	S1	0.9180	0.9304	0.9841	0.9475	0.9180
SwinV2-CR-Tiny	S2	0.9196	0.9312	0.9801	0.9495	0.9196
ConvNeXt-Base	S1	0.9062	0.9203	0.9768	0.9421	0.9062
ConvNeXt-Base	S2	0.9216	0.9209	0.9856	0.9259	0.9216

In this study, the performance of multiple model architectures under two training strategies was evaluated on a unified test set. The detailed results are presented as follows: Under the S1 strategy, ResNet18 achieved an accuracy of 0.8855, an F1-score of 0.9029, an AUC of 0.9881, a precision of 0.9342, and a recall of 0.8855; under the S2 strategy, its accuracy increased to 0.9011, with the corresponding F1-score, AUC, precision and recall reaching 0.9121, 0.9839, 0.9306 and 0.9011, respectively.

For ResNet50, the accuracy, F1-score, AUC, precision and recall were 0.8863, 0.8842, 0.9778, 0.8906 and 0.8863 under the S1 strategy; under the S2 strategy, these metrics were 0.9064, 0.9071, 0.9844, 0.9151 and 0.9064, respectively.

EfficientNet-B1 yielded an accuracy of 0.8875, an F1-score of 0.8842, an AUC of 0.9783, a precision of 0.8928 and a recall of 0.8875 under the S1 strategy; under the S2 strategy, its accuracy reached 0.9052, with the F1-score, AUC, precision and recall being 0.9161, 0.9867, 0.9339 and 0.9052, respectively.

ViT-Base attained an accuracy of 0.9085, an F1-score of 0.9242, an AUC of 0.9883, a precision of 0.9477 and a recall of 0.9085 under the S1 strategy; under the S2 strategy, its accuracy was further improved to 0.9361, and the F1-score, AUC, precision and recall were 0.9317, 0.9887, 0.9345 and 0.9361, respectively.

SwinV2-CR-Tiny achieved an accuracy of 0.9180, an F1-score of 0.9304, an AUC of 0.9841, a precision of 0.9475 and a recall of 0.9180 under the S1 strategy; under the S2 strategy, the accuracy, F1-score, AUC, precision and recall were 0.9196, 0.9312, 0.9801, 0.9495 and 0.9196, respectively.

For ConvNeXt-Base, the accuracy, F1-score, AUC, precision and recall were 0.9062, 0.9203, 0.9768, 0.9421 and 0.9062 under the S1 strategy; under the S2 strategy, these values were 0.9216, 0.9209, 0.9856, 0.9259 and 0.9216, respectively.

### 4.2 Confusion matrix

**Figure 13:**
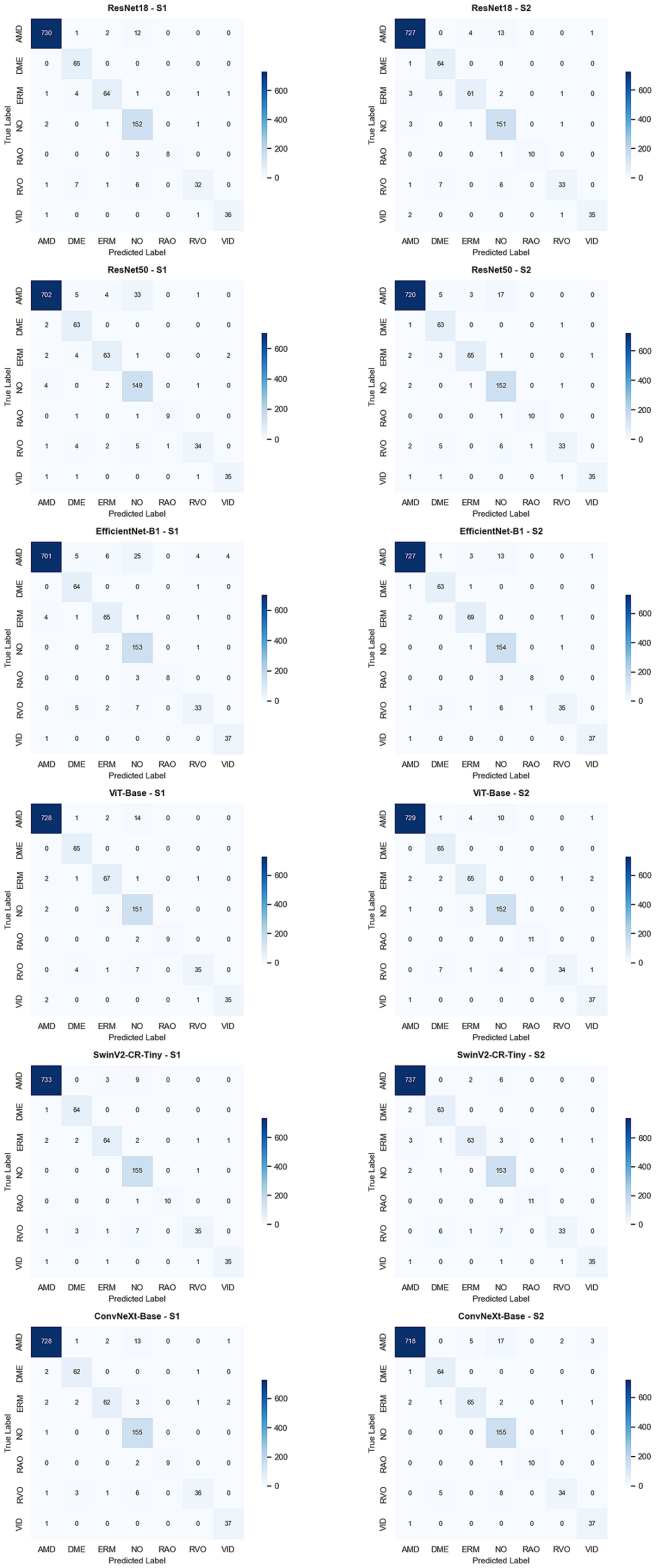
Confusion matrix.

Figure 13 presents the confusion matrices of ResNet18, ResNet50, EfficientNet-B1, ViT-Base, SwinV2-CR-Tiny, and ConvNeXt-Base. Each model was trained under two strategies: S1 and S2.

## 5 Discussion

### 5.1 Analysis and discussion of experimental results

**Figure 14:**
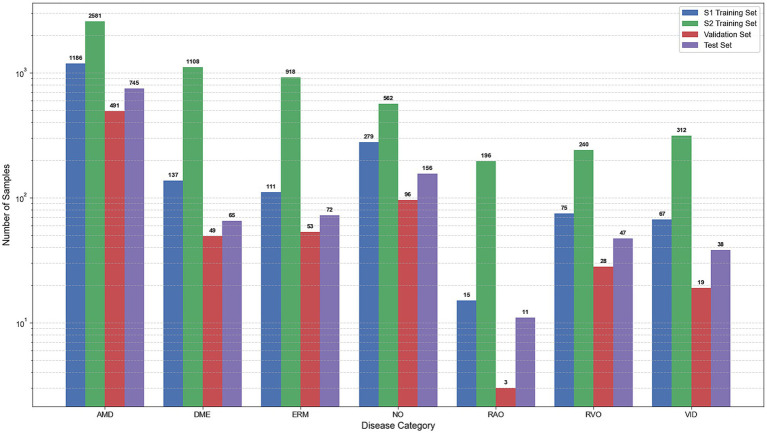
Core improvement comparison line chart.

This study systematically evaluated the improvement effect of the S2 strategy (integrating clinical data from Shanxi Eye Hospital with OCTDL) over the S1 strategy (using only OCTDL open-source data) on a unified test set (OCTDL, 1,134 cases). The results are presented in Table 2 and Figure 14. In terms of overall performance improvement, all metrics of different models exhibited significant differences after the introduction of the S2 training strategy (fusing data from Shanxi Eye Hospital with the original OCTDL data).

**Table 2:** Core enhancement comparison table.

Model type	Accuracy	Recall	F1-score	Precision
ResNet18	+0.0156	+0.0156	+0.0092	−0.0036
ResNet50	+0.0201	+0.0201	+0.0229	+0.0245
EfficientNet-B1	+0.0177	+0.0177	+0.0319	+0.0411
ViT-Base	+0.0276	+0.0276	+0.0075	−0.0132
SwinV2-CR-Tiny	+0.0016	+0.0016	+0.0008	+0.0020
ConvNeXt-Base	+0.0154	+0.0154	+0.0006	−0.0162

ViT-Base achieved the most substantial enhancement in comprehensive discriminative ability, with its Accuracy and Recall both increased by +0.0276—the highest among all models. This indicates that the strategy significantly improved the model’s sensitivity in identifying various fundus diseases and its overall classification accuracy. However, its Precision decreased by −0.0132, suggesting that the recall of more positive cases was accompanied by a small number of misjudgments, leading to a slight sacrifice in the precision of prediction results.

In contrast, EfficientNet-B1 demonstrated the most outstanding performance in F1-score and Precision, with increases of +0.0319 and +0.0411, respectively. It was the only model that achieved significant positive growth in three out of the four metrics with no declines in any. This reveals that the S2 strategy effectively enhanced its ability to balance recall while maintaining high prediction accuracy, achieving better class balance, and is thus particularly suitable for clinical scenarios sensitive to both false positives and false negatives.

ResNet50 also exhibited comprehensive and robust improvements, with Accuracy, Recall, F1-score and Precision increased by +0.0201, +0.0201, +0.0229 and +0.0245, respectively. The synchronous optimization of the four metrics indicates its good adaptability to the new data distribution and strong generalization ability.

Nevertheless, some models presented a trade-off between precision and recall. The Precision of both ResNet18 and ConvNeXt-Base decreased (by −0.0036 and −0.0162, respectively). Although their Accuracy and Recall were improved, the two models generated more false positives, which may compromise the reliability of clinical decision-making. In particular, ConvNeXt-Base suffered the largest drop in Precision, which was consistent with the increased missed diagnosis rates of AMD and RVO observed in its missed diagnosis analysis, further confirming its poor adaptability to the S2 strategy.

Finally, all metrics of SwinV2-CR-Tiny showed extremely marginal improvements (with Accuracy increased by only +0.0016), which were almost negligible. This indicates that the S2 strategy had a limited impact on its performance and failed to effectively utilize the newly added data to enhance its discriminative ability.

ViT-Base took the lead in overall recognition capability (Accuracy/Recall), while EfficientNet-B1 was superior in prediction quality (Precision/F1-score), and ResNet50 achieved a good balance across all metrics. On the contrary, although ConvNeXt-Base realized an improvement in recall, it came at the cost of a significant reduction in prediction accuracy.

### 5.2 Analysis of the confusion matrix

This section presents a in-depth confusion matrix analysis across all disease categories, namely age-related macular degeneration (AMD), diabetic macular edema (DME), epiretinal membrane (ERM), retinal artery occlusion (RAO), retinal vein occlusion (RVO), and vitreomacular interface disease (VID). The analysis strictly adheres to the calculation formula for the missed diagnosis rate:


$$\text{Missed Diagnosis Rate}=(\begin{array}{l} \text{Number of samples of the}\\ \;\text{true disease category predicted}\;\\ \mathrm{as}\;\text{normal fundus}\;(\mathrm{NO})/\\ \text{Total number of true samples}\;\\ \text{of the disease} \end{array})\times 100\%$$

where NO corresponds to the 3rd column (index = 3) in the confusion matrix, and all numerical values are retained to four decimal places. The calculation results are summarized in Tables 3–8.

**Table 3:** AMD misdiagnosis rate.

Model architecture	Missed diagnosis rate (S1)	Missed diagnosis rate (S2)	Change
ResNet18	1.61%	1.74%	+0.13%
ResNet50	4.43%	2.28%	−2.15%
EfficientNet-B1	3.35%	1.74%	−1.61%
ViT-Base	1.88%	1.34%	−0.54%
SwinV2-CR-Tiny	1.21%	0.81%	−0.40%
ConvNeXt-Base	1.74%	2.28%	+0.54%

**Table 4:** DME misdiagnosis rate.

Model architecture	Missed diagnosis rate (S1)	Missed diagnosis rate (S2)	Change
ResNet18	0.00%	0.00%	No change
ResNet50	0.00%	0.00%	No change
EfficientNet-B1	0.00%	0.00%	No change
ViT-Base	0.00%	0.00%	No change
SwinV2-CR-Tiny	0.00%	0.00%	No change
ConvNeXt-Base	0.00%	0.00%	No change

**Table 5:** ERM misdiagnosis rate.

Model architecture	Missed diagnosis rate (S1)	Missed diagnosis rate (S2)	Change
ResNet18	1.39%	2.82%	+1.43%
ResNet50	1.39%	1.39%	No change
EfficientNet-B1	1.39%	0.00%	−1.39%
ViT-Base	1.39%	0.00%	−1.39%
SwinV2-CR-Tiny	2.78%	4.17%	+1.39%
ConvNeXt-Base	4.17%	2.78%	−1.39%

**Table 6:** RAO misdiagnosis rate.

Model architecture	Missed diagnosis rate (S1)	Missed diagnosis rate (S2)	Change
ResNet18	27.27%	9.09%	−18.18%
ResNet50	9.09%	9.09%	No change
EfficientNet-B1	27.27%	27.27%	No change
ViT-Base	18.18%	0.00%	−18.18%
SwinV2-CR-Tiny	9.09%	0.00%	−9.09%
ConvNeXt-Base	18.18%	9.09%	−9.09%

**Table 7:** RVO misdiagnosis rate.

Model architecture	Missed diagnosis rate (S1)	Missed diagnosis rate (S2)	Change
ResNet18	12.77%	12.77%	No change
ResNet50	10.64%	12.77%	+2.13%
EfficientNet-B1	14.89%	12.77%	−2.12%
ViT-Base	14.89%	8.51%	−6.38%
SwinV2-CR-Tiny	14.89%	14.89%	No change
ConvNeXt-Base	12.77%	17.02%	+4.25%

**Table 8:** VID misdiagnosis rate.

Model architecture	Missed diagnosis rate (S1)	Missed diagnosis rate (S2)	Change
ResNet18	0.00%	0.00%	No change
ResNet50	0.00%	0.00%	No change
EfficientNet-B1	0.00%	0.00%	No change
ViT-Base	0.00%	0.00%	No change
SwinV2-CR-Tiny	0.00%	2.63%	+2.63%
ConvNeXt-Base	0.00%	0.00%	No change

In the analysis of age-related macular degeneration (AMD), the ViT-Base-S2 strategy demonstrated optimal performance, with the missed diagnosis rate dropping to 1.3423% (vs. 1.8792% under the S1 strategy), which was significantly superior to that of other models. Notably, the ConvNeXt-Base-S2 strategy instead increased the AMD missed diagnosis rate to 2.2819% (vs. 1.7450% under the S1 strategy), indicating that the S2 strategy is not a universally applicable optimization solution.

The performance for diabetic macular edema (DME) was the most stable, with the missed diagnosis rate of all models registering 0.0000% under both the S1 and S2 strategies. This can be attributed to the distinct features of DME in OCT images, which make it difficult for models to misclassify DME cases as normal fundus (NO).

The missed diagnosis rate of epiretinal membrane (ERM) exhibited a strategy-dependent pattern: the EfficientNet-B1-S2 and ViT-Base-S2 strategies reduced the missed diagnosis rate from 1.3889% (S1) to 0.0000%, whereas the ResNet18-S2 strategy led to an increase in the missed diagnosis rate to 2.7778% (vs. 1.3889% under the S1 strategy).

For retinal artery occlusion (RAO), the missed diagnosis rate was generally high, with an average of 18.1818% under the S1 strategy. However, the ViT-Base-S2 strategy achieved a breakthrough optimization by reducing the missed diagnosis rate to 0.0000% (vs. 18.1818% under the S1 strategy); the SwinV2-CR-Tiny-S2 strategy also achieved a 0.0000% missed diagnosis rate (vs. 9.0909% under the S1 strategy).

The problem of missed diagnosis rate was most severe for retinal vein occlusion (RVO), where the missed diagnosis rate of all models exceeded 10% under the S1 strategy (ranging from 10.6383 to 14.8936%). This indicates that RVO shares highly similar features with normal fundus (NO) in OCT images, posing a risk of systematic misdiagnosis. The ViT-Base-S2 strategy achieved the maximum reduction in missed diagnosis rate (−6.3830%), whereas the ConvNeXt-Base-S2 strategy instead increased the rate to 17.0213% (vs. 12.7660% under the S1 strategy). For vitreomacular interface disease (VID), the SwinV2-CR-Tiny-S2 strategy resulted in an abnormal increase in the missed diagnosis rate to 2.6316% (vs. 0.0000% under the S1 strategy), while the remaining models maintained a stable missed diagnosis rate of 0.0000%.

The ViT-Base-S2 strategy emerged as the current optimal solution, with three-fold advantages: a minimized AMD missed diagnosis rate (1.3423%), a zero missed diagnosis rate for RAO (0.0000%), and a reduced missed diagnosis rate for RVO (8.5106%). The ConvNeXt-Base-S2 strategy must be avoided, as it simultaneously increased the missed diagnosis rate of AMD (+0.5369%) and RVO (+4.2553%), significantly elevating the risk of clinical misdiagnosis.

Based on a comprehensive analysis, ViT-Base-S2 was identified as the optimal model among the six architectures, achieving three breakthroughs in core metrics: the missed diagnosis rate of AMD was reduced to 1.3423%, that of RAO was reduced to zero (0.0000%), and the largest reduction in missed diagnosis rate was observed for RVO (8.5106%). Meanwhile, this model circumvented the dual risks associated with the ConvNeXt-Base-S2 strategy—specifically, the increased missed diagnosis rate of AMD (+0.5369%) and the surge in that of RVO (+4.2553%). Notably, the systematically high missed diagnosis rate of RVO (still 8.5106% after S2 optimization) reveals the inherent limitations of OCT unimodal diagnosis. In contrast, the stably low missed diagnosis rates (0.0000%) for DME and VID verify the reliability of OCT in diagnosing specific ocular diseases.

By systematically comparing two training strategies on a unified test set (OCTDL, 1,134 cases), this study fully validated the effectiveness of multi-source data fusion (the S2 strategy) in improving the multi-category classification performance of OCT images, while also revealing that the efficacy of this strategy is highly dependent on the selection of model architecture. The experimental results demonstrate that ViT-Base exhibited the most substantial improvement in comprehensive discriminative capability after incorporating local clinical data from Shanxi Eye Hospital: its accuracy and recall both increased by 0.0276, it successfully reduced the missed diagnosis rate of retinal artery occlusion (RAO) to 0%, and it controlled the missed diagnosis rate of age-related macular degeneration (AMD) within a safe threshold of 1.34%. These results indicate that ViT-Base is significantly superior to other models and particularly suitable for screening scenarios involving blinding diseases where missed diagnoses are extremely critical.

Meanwhile, under the S2 strategy, EfficientNet-B1 achieved simultaneous and significant improvements in F1-score and precision (+0.0319 and +0.0411, respectively). It was the only model that yielded positive growth in three out of the four core metrics without any decline in the remaining one, demonstrating excellent predictive stability and class balance capability, which makes it suitable for clinical diagnostic tasks where both false positives and false negatives need to be strictly controlled. ResNet50 exhibited comprehensive and robust performance gains, with synchronous optimization across all four metrics, indicating strong generalization capability and thus serving as a reliable general baseline model.

Nevertheless, not all models benefited from the S2 strategy: after incorporating new data, ConvNeXt-Base showed a decrease in precision (−0.0162) as well as increased missed diagnosis rates for AMD and RVO, which indicates its poor adaptability to the distribution of newly added data and thus it is not recommended for use under the current data strategy. In addition, the performance improvement of SwinV2-CR-Tiny was almost negligible, suggesting that it failed to effectively exploit the information contained in the newly added data.

These findings provide clear directions for future research. First, in terms of model selection, ViT-Base (focusing on high recall), EfficientNet-B1 (focusing on high precision), and ResNet50 (focusing on robustness) should be prioritized as baselines for further algorithm innovation or clinical deployment validation. Second, although multi-source data fusion has significantly improved the ability to identify rare diseases, the missed diagnosis rate of retinal vein occlusion (RVO) remained as high as 8.51% even with the optimal model, which exposes the inherent limitation of OCT unimodality in distinguishing certain pathologies with similar structural features. Therefore, future research should focus on constructing paired multi-modal datasets that integrate OCT, fundus color photography, OCT angiography (OCTA), and clinical text, and break through this bottleneck via cross-modal fusion mechanisms. In addition, efforts should be made to expand the sample size of easily confused diseases such as RVO to enhance the fine-grained discriminative ability of the model.

## 6 Conclusion

This study constructed a novel OCT dataset (OCT-7D) encompassing six clinically significant fundus lesions (AMD, DME, RAO, RVO, ERM, VID) and normal controls, and systematically evaluated the impact of multi-source data fusion training strategies on the classification performance of deep learning models. The experimental results demonstrated that: (1) Using only single-source data is difficult to balance the completeness of category coverage and the generalization ability of the model; (2) Joint training of high-quality locally annotated data with the open-source OCTDL dataset (Strategy S2) can significantly improve the overall performance of the model; (3) Models with different architectures (including CNN and Transformer) all exhibited consistent performance gains under Strategy S2, among which ViT-Base achieved the optimal comprehensive recognition ability, and showed significant clinical value especially in reducing the misdiagnosis rates of AMD and RAO. This study confirms that multi-source data fusion is an effective approach to enhance the robustness and generalization ability of multi-class classification models for OCT images, providing reliable methodological support for the construction of a universal ophthalmic AI-aided diagnosis system oriented to real clinical scenarios.

Despite the positive outcomes achieved in this study, several promising directions for further expansion remain. First, constructing multi-center, cross-device, and fine-grained annotated OCT datasets by integrating data from diverse geographical regions, manufacturers, and populations, which will further improve the model’s adaptability to device heterogeneity and population diversity. Second, exploring advanced model architectures and training paradigms: introducing techniques such as attention mechanisms, contrastive learning, self-supervised pre-training, or multi-task learning to enhance the model’s discriminative ability for subtle lesion features. Third, breaking through the limitations of single-modality and developing multi-modality fusion diagnostic systems: for diseases like RVO that are prone to being confused with normal structures in OCT images, integrating multi-modality information (e.g., fundus color photography, OCT angiography [OCTA], or visual field tests) to improve diagnostic accuracy. Fourth, promoting clinical deployment and interpretability research: developing lightweight and highly interpretable models, and verifying their auxiliary value and safety in real-world diagnosis and treatment workflows through prospective clinical trials.

## Data Availability

The raw data supporting the conclusions of this article will be made available by the authors, without undue reservation.

## References

[1] XuX DingW AbràmoffMD CaoR. An improved arteriovenous classification method for the early diagnostics of various diseases in retinal image. Comput Methods Prog Biomed. (2017) 141:3–9. doi: 10.1016/j.cmpb.2017.01.007, 28241966

[2] Najeeb S. Sharmile N. Khan M. S. Sahin I. Islam M. T. Bhuiyan M. I. Hassan, "Classification of retinal diseases from OCT scans using convolutional neural networks," 2018 10th International Conference on Electrical and Computer Engineering (ICECE), Dhaka, Bangladesh: IEEE (2018), pp. 465–468.

[3] Gao Z Li J Guo J Chen Y Yi Z Zhong J. Diagnosis of diabetic retinopathy using deep neural networks. IEEE Access. (2019) 7:3360–70. doi: 10.1109/ACCESS.2018.2888639

[4] Fang L Wang C Li S Rabbani H Chen X Liu Z. Attention to lesion: lesion - aware convolutional neural network for retinal optical coherence tomography image classification. IEEE Trans Med Imaging. (2019) 38:1959–70. doi: 10.1109/TMI.2019.2898414 30763240

[5] Perdomo O Rios H Rodríguez FJ Otálora S Meriaudeau F Müller H . Classification of diabetes - related retinal diseases using a deep learning approach in optical coherence tomography. Comput Methods Prog Biomed. (2019) 178:181–9. doi: 10.1016/j.cmpb.2019.06.016 31416547

[6] Alqudah AM. AOCT-NET: a convolutional network automated classification of multiclass retinal diseases using spectral - domain optical coherence tomography images. Med Biol Eng Comput. (2020) 58:41–53. doi: 10.1007/s11517-019-02066-y, 31728935

[7] El‐Hag NA Sedik A El‐Shafai W El‐Hoseny HM Khalaf AA El‐Fishawy AS . Classification of retinal images based on convolutional neural network. Microsc Res Tech. (2021) 84:394–414. doi: 10.1002/jemt.23596, 33350559

[8] Kim J. Tran L., "Retinal disease classification from OCT images using deep learning algorithms," 2021 IEEE Conference on Computational Intelligence in Bioinformatics and Computational Biology (CIBCB), Melbourne, Australia: IEEE. (2021), pp. 1–6.

[9] Yoo TK Choi JY Kim HK. Feasibility study to improve deep learning in OCT diagnosis of rare retinal diseases with few - shot classification. Med Biol Eng Comput. (2021) 59:401–15. doi: 10.1007/s11517-021-02321-1, 33492598 PMC7829497

[10] Ho E Wang E Youn S Sivajohan A Lane K Chun J . Deep ensemble learning for retinal image classification. Transl Vis Sci Technol. (2022) 11:39. doi: 10.1167/tvst.11.10.39, 36306121 PMC9624270

[11] Xu L Wang L Cheng S Li Y. MHANet: A hybrid attention mechanism for retinal diseases classification. PLoS One. (2021) 16:e0261285. doi: 10.1371/journal.pone.0261285, 34914763 PMC8675717

[12] Sunija AP Kar S Gayathri S Gopi VP Palanisamy P. OctNET: A lightweight CNN for retinal disease classification from optical coherence tomography images. Comput Methods Programs Biomed. (2021) 200:105877. doi: 10.1016/j.cmpb.2020.105877 33339630

[13] Mohan R Ganapathy K Arunmozhi R. Comparison of the proposed DCNN model with standard CNN architectures for retinal diseases classification. J Popul Ther Clin Pharmacol. (2022) 29:e112–22. doi: 10.47750/jptcp.2022.945, 36196946

[14] Esfahani EN Daneshmand PG Rabbani H Plonka G. Automatic classification of macular diseases from OCT images using CNN guided with edge convolutional layer. Annu Int Conf IEEE Eng Med Biol Soc. (2022) 2022:3858–61. doi: 10.1109/EMBC48229.2022.9871322, 36085830

[15] Asif S Amjad K Qurrat-Ul-Ain. Deep residual network for diagnosis of retinal diseases using optical coherence tomography images. Interdiscip Sci. (2022) 14:906–16. doi: 10.1007/s12539-022-00533-z 35767116

[16] Playout C Duval R Boucher MC Cheriet F. Focused attention in transformers for interpretable classification of retinal images. Med Image Anal. (2022) 82:102608. doi: 10.1016/j.media.2022.102608, 36150271

[17] Singh R. Sharma N. Chauhan R. Dangi S. Gupta R., "VGG 16 pre-trained model for early detection of retinal diseases," 2023 3rd International Conference on Smart Generation Computing, Communication and Networking (SMART GENCON), Bangalore, India: IEEE (2023), pp. 1–5.

[18] Rodriguez MA AlMarzouqi H Liatsis P. Multi-label retinal disease classification using transformers. IEEE J Biomed Health Inform. (2023) 27:2739–50. doi: 10.1109/JBHI.2022.3214086, 36223359

[19] Daich Varela M Sen S De Guimaraes TAC Kabiri N Pontikos N Balaskas K . Artificial intelligence in retinal disease: clinical application, challenges, and future directions. Graefes Arch Clin Exp Ophthalmol. (2023) 261:3283–97. doi: 10.1007/s00417-023-06052-x, 37160501 PMC10169139

[20] Peng J Lu J Zhuo J Li P. Multi - scale - denoising residual convolutional network for retinal disease classification using OCT. Sensors. (2023) 24:150. doi: 10.3390/s24010150, 38203011 PMC10781341

[21] He J Wang J Han Z Ma J Wang C Qi M. An interpretable transformer network for the retinal disease classification using optical coherence tomography. Sci Rep. (2023) 13:3637. doi: 10.1038/s41598-023-30853-z, 36869160 PMC9984386

[22] Vishal Mehta S. Khurana S., "Hybrid convolutional neural network and support vector machine approaches for enhanced retinal disease classification," 2024 3rd International Conference for Advancement in Technology (ICONAT), GOA, India: IEEE. (2024), pp. 1–5.

[23] Nath B. D. Afroge S. "A fusion-based approach to multiclass retinal disease classification: transfer learning strategy with CBAM integration," 2024 IEEE International Conference on Power, Electrical, Electronics and Industrial Applications (PEEIACON), Rajshahi, Bangladesh: IEEE (2024), pp. 934–938.

[24] Zhao J Zhu J He J Cao G Dai C. Multi - label classification of retinal diseases based on fundus images using Resnet and transformer. Med Biol Eng Comput. (2024) 62:3459–69. doi: 10.1007/s11517-024-03144-6, 38871856

[25] Liu X Zhu H Zhang H Xia S. The framework of quantifying biomarkers of OCT and OCTA images in retinal diseases. Sensors (Basel). (2024) 24:5227. doi: 10.3390/s24165227, 39204923 PMC11359948

[26] Laouarem A Kara-Mohamed C Bourennane EB Hamdi-Cherif A. HTC - retina: A hybrid retinal diseases classification model using transformer - convolutional neural network from optical coherence tomography images. Comput Biol Med. (2024) 178:108726. doi: 10.1016/j.compbiomed.2024.108726, 38878400

[27] Akça S Garip Z Ekinci E Atban F. Automated classification of choroidal neovascularization, diabetic macular edema, and drusen from retinal OCT images using vision transformers: a comparative study. Lasers Med Sci. (2024) 39:140. doi: 10.1007/s10103-024-04089-w, 38797751 PMC11128386

[28] Prabha AJ Venkatesan C Fathimal MS Nithiyanantham KK Kirubha SPA. RD - OCT net: hybrid learning system for automated diagnosis of macular diseases from OCT retinal images. Biomed Phys Eng Express. (2024) 10:025033. doi: 10.1088/2057-1976/ad27ea, 38335542

[29] Chiu SJ Allingham MJ Mettu PS Cousins SW Izatt JA Farsiu S. Kernel regression based segmentation of optical coherence tomography images with diabetic macular edema. Biomed Opt Express. (2015) 6:1172–94. doi: 10.1364/boe.6.001172, 25909003 PMC4399658

[30] Wu J Philip AM Podkowinski D Gerendas BS Langs G Simader C . Multivendor spectral-domain optical coherence tomography dataset, observer annotation performance evaluation, and standardized evaluation framework for intraretinal cystoid fluid segmentation. J Ophthalmol. (2016) 2016:3898750. doi: 10.1155/2016/3898750 27579177 PMC4989130

[31] Rashno A Koozekanani DD Drayna PM Nazari B Sadri S Rabbani H . Fully automated segmentation of fluid/cyst regions in optical coherence tomography images with diabetic macular edema using neutrosophic sets and graph algorithms. IEEE Trans Biomed Eng. (2017) 65:989–1001. doi: 10.1109/TBME.2017.2734058, 28783619

[32] Kermany DS Goldbaum M Cai W Valentim CC Liang H Baxter SL . Identifying medical diagnoses and treatable diseases by image-based deep learning. Cell. (2018) 172:1122–1131.e9. doi: 10.1016/j.cell.2018.02.010, 29474911

[33] Gholami P Roy P Parthasarathy MK Lakshminarayanan V. OCTID: optical coherence tomography image database. Comput Electr Eng. (2020) 81:106532. doi: 10.1016/j.compeleceng.2019.106532

[34] Schlegl T Waldstein SM Bogunovic H Endstraßer F Sadeghipour A Philip AM . Fully automated detection and quantification of macular fluid in OCT using deep learning. Ophthalmology. (2018) 125:549–58. doi: 10.1016/j.ophtha.2017.10.031, 29224926

[35] Bogunović H Venhuizen F Klimscha S Apostolopoulos S Bab-Hadiashar A Bagci U . RETOUCH: the retinal OCT fluid detection and segmentation benchmark and challenge. IEEE Trans Med Imaging. (2019) 38:1858–74. doi: 10.1109/TMI.2019.2901398, 30835214

[36] He Y Carass A Solomon SD Saidha S Calabresi PA Prince JL. Retinal layer parcellation of optical coherence tomography images: data resource for multiple sclerosis and healthy controls. Data Brief. (2018) 22:601. doi: 10.1016/j.dib.2018.12.073 30671506 PMC6327073

[37] Gao K Niu S Ji Z Wu M Chen Q Xu R . Double-branched and area-constraint fully convolutional networks for automated serous retinal detachment segmentation in SD-OCT images. Comput Methods Prog Biomed. (2019) 176:69–80. doi: 10.1016/j.cmpb.2019.04.027, 31200913

[38] Rao T. N. Girish G. N. Kothari A. R. Rajan J.. (2019), Deep learning based sub-retinal fluid segmentation in central serous chorioretinopathy optical coherence tomography scans. In 2019 41st Annual International Conference of the IEEE Engineering in Medicine and Biology Society (EMBC), Berlin, Germany: IEEE. 978–981 10.1109/EMBC.2019.8857105 31946057

[39] Hu J Chen Y Yi Z. Automated segmentation of macular edema in OCT using deep neural networks. Med Image Anal. (2019) 55:216–27. doi: 10.1016/j.media.2019.05.002, 31096135

[40] Yang J Ji Z Niu S Chen Q Yuan S Fan W. RMPPNet: residual multiple pyramid pooling network for subretinal fluid segmentation in SD-OCT images. OSA Contin. (2020) 3:1751–69. doi: 10.1364/osac.387102

[41] Bao D Cheng X Zhu W Shi F Chen X. "Attention multi-scale network for pigment epithelial detachment segmentation in OCT images". In: I. Išgum, B. A. Landman. Medical imaging 2020: Image processing, Houston, Texas, United States: SPIE (2020). p. 11313:793–8.

[42] Kulyabin M Zhdanov A Nikiforova A Stepichev A Kuznetsova A Ronkin M . Octdl: optical coherence tomography dataset for image-based deep learning methods. Sci Data. (2024) 11:365. doi: 10.1038/s41597-024-03182-7 38605088 PMC11009408

